# Clinical Oncological Tolerance of Testosterone Replacement Therapy Over Two Years Among Patients With High-Risk or Very High-Risk Prostate Cancer Undergoing Radiotherapy

**DOI:** 10.7759/cureus.92806

**Published:** 2025-09-20

**Authors:** Kazuyoshi Shigehara, Renato Naito, Tetsuya Kawahara, Yuki Kato, Rei Shinzawa, Hiroshi Yaegshi, Takahiro Nohara, Kouji Izumi, Atsushi Mizokami

**Affiliations:** 1 Integrative Cancer Therapy and Urology, Kanazawa University Graduate School of Medical Science, Kanazawa, JPN; 2 Urololgy, Fukui-ken Saiseikai Hospital, Fukui, JPN

**Keywords:** hypogonadism in men, prostate cancer, radiotherapy (rt), testosterone replacement, testosterone (tt)

## Abstract

Objective: Studies have demonstrated that testosterone replacement therapy (TRT) for patients with prostate cancer following curative local therapy does not significantly increase the biochemical and clinical recurrences of cancer. However, evidence on oncological tolerance of TRT for patients with high-risk prostate cancer is currently limited. This study assessed clinical oncological tolerance of TRT in patients with high- or very high-risk prostate cancer undergoing radiotherapy.

Methods: Patients with serum total testosterone (TT) levels <300 ng/dl and any hypogonadal symptoms were screened. Cases without definitive evidence of prostate cancer recurrence were considered as candidates for TRT based on serum prostate-specific antigen (PSA) levels below 0.2 ng/ml for more than 2 years after completing all radical treatment. All patients received testosterone enanthate intramuscularly every four weeks. Blood biochemistry, including PSA levels, was evaluated every three months after TRT. Serum TT levels were measured every six months. Radiological imaging was performed every year.

Results: A total of 20 patients were included in the study. At TRT initiation, the mean age of the patients was 74.1 years. The mean TT and PSA levels were 0.608 and 0.021 ng/ml, respectively. TRT was continued for a mean of 43.7 (3-132) months. Twelve cases (63%) could complete the two-year TRT. The serum PSA levels showed a significant increase for six months after TRT (p < 0.05) and did not change significantly until 24 months. The serum TT levels increased significantly at the six-month visit and remained stable until the 24th month. The hemoglobin levels significantly increased by the sixth month but remained unchanged thereafter. No cases showed biochemical and clinical recurrences of prostate cancer.

Conclusion: TRT for two years might be tolerated for cancer control among patients with high-risk prostate cancer who had undergone radiotherapy. Since this was a retrospective study without a control group, further prospective studies, including a large number of subjects and a control group, are required to reach a more definite conclusion.

## Introduction

A decline in serum testosterone levels is significantly associated with sexual dysfunction, decrease in muscle volume and bone mineral density, metabolic syndrome, and menopausal symptoms [1,2]. These signs and symptoms caused by age-related decline in testosterone levels are known as symptoms of late-onset hypogonadism (LOH) syndrome [3]. Testosterone replacement therapy (TRT) is a useful approach to improve the various conditions resulting from testosterone deficiency [4].

The incidence of prostate cancer increases with an aging population, and hormonal therapy (e.g., androgen deprivation therapy (ADT)) is being implemented as a treatment. ADT is used to treat advanced prostate cancer and as neoadjuvant and adjuvant therapy for various radiotherapies [5]. However, failure to recover total testosterone (TT) levels after ADT completion is an issue. One study demonstrated that 18.4% of patients had low TT levels (<3.0 ng/ml) two years after the cessation of a six-month neoadjuvant ADT before low-dose rate brachytherapy, and the median interval for recovering to normal TT was 15 months [6]. Another study, which included patients with long-term (median: 17 months) adjuvant ADT after radical prostatectomy, found that serum TT levels did not recover to the normal range in 45% and 25% of patients at 12 and 36 months, respectively, after completing ADT [7].

ADT causes various side effects that are similar to the clinical conditions of LOH syndrome. Moreover, it can result in a decreased quality of life (QOL) for patients with prostate cancer [8]. The overall survival rate from prostate cancer is currently increasing due to improvements in its treatment, making the maintenance of QOL an important issue, especially in patients who underwent curative prostate cancer treatment. Although TRT is useful in improving the symptoms caused by testosterone deficiency, it is generally contraindicated for patients with a history of prostate cancer. In clinical practice, there is consensus that ADT can clinically and biochemically improve prostate cancer, leading to the belief that testosterone administration can accelerate prostate cancer progression [9]. However, exogenous testosterone supplementation beyond the androgen-androgen receptor binding saturation concentration has little additional effect [10]. This proves that TRT has potential as a new treatment option for patients with hypogonadism and a prostate cancer history.

As described above, several studies have demonstrated that TRT for patients with prostate cancer following curative local therapy does not significantly increase the biochemical and clinical recurrences of cancer. However, evidence on oncological tolerance of TRT for patients with high-risk prostate cancer is currently limited. This retrospective observational study assessed the clinical oncological tolerance of TRT, specifically the rate of biochemical recurrence, in patients with high- or very high-risk prostate cancer who underwent radiotherapy.

## Materials and methods

This was a single-center, retrospective observational study conducted at Kanazawa University Hospital, Kanazawa, Japan. This study was approved by the Medical Ethics Committee of Kanazawa University (approval number: 2934-3) and performed in accordance with the Ethical Guidelines for Medical and Health Research.

Study population

Twenty patients with high- or very high-risk prostate cancer, who were cured by radiotherapy and subsequently underwent TRT between 2012 and 2024, were included in the present analysis. All patients received high-dose rate (HDR) brachytherapy and/or external beam radiotherapy (EBRT) after neoadjuvant-combined androgen blockade (CAB) therapy for six months following our institution's protocol [11]. Adjuvant CAB therapy was continued for two years after radiotherapy.

Prostate cancer risk is defined as follows based on the National Comprehensive Cancer Network classification (2019, version 2) [12]: ‘high-risk’ as T3a disease, Gleason score ≥ 8, or prostate specific antigen (PSA) levels > 20 ng/ml; and ‘very high-risk’ as T3b or T4 disease or primary Gleason pattern 5 or >5 scores with histological grade group 4 or 5.

Criteria for TRT

TRT was administered to patients who met the following criteria: those with serum TT levels below 300 ng/dl, those with any hypogonadal symptoms, those with serum PSA levels < 0.2 ng/ml more than two years after cancer treatment (adjuvant ADT was finished), those without definitive findings based on radiological imaging, and those who wanted TRT. Hypogonadal symptoms were assessed by each attending physician based on the patients’ subjective complaints. Patients diagnosed with a Stage IV disease at initial presentation, those with unstable and poor cancer control, as determined by each attending physician, and those who did not want TRT, were excluded from TRT administration. 

TRT was administered to patients who requested the treatment after their written informed consent was obtained.

TRT protocol and follow-up

The patients received testosterone enanthate (TE) (in principle, 250 mg; Enarmon Depot®; ASKA Pharmaceutical Co., Ltd., Tokyo, Japan) intramuscularly every four weeks. Blood biochemistry, including the PSA levels, was evaluated every three months after TRT, and serum TT levels were measured every six months. In addition, radiological imaging studies were performed every year. TRT was interrupted in patients with PSA elevations of 2.0 ng/ml from the nadir, disease progression based on radiological examinations, any adverse events associated with TRT (e.g., polycythemia), and no definite improvement in hypogonadal symptoms.

Data analysis

Patients with a two-year treatment completion were enrolled in the analysis. The hemoglobin (Hb) values and the PSA levels measured every three months, and the serum TT levels every 6 months after treatment, were extracted to assess the oncological tolerance and the biochemical effects of the 24-month TRT. The primary endpoint was serum PSA levels after TRT. The secondary endpoint included Hb values and serum TT levels after TRT, adverse events, and cardiovascular or thromboembolic events.

The variables from the baseline to the 24th month visit were compared using Wilcoxon’s signed rank test. Statistical analyses were performed using IBM SPSS Statistics for Windows, version 25 (IBM Corp., Armonk, New York, United States). In all analyses, a p-value < 0.05 was taken to indicate statistical significance.

## Results

Demographic details

Table 1 presents the background of the patients before prostate cancer treatment. Very high-risk prostate cancer was observed in seven (35.0%) cases, and lymph node metastasis was observed in two cases. At TRT initiation, the mean age (range) of the 20 patients was 74.1 years (range, 67-85). The mean TT and PSA levels were 0.608 (range, 0.03-2.29) and 0.021 (range, <0.006-0.167) ng/ml, respectively. The mean duration from the end of radiotherapy to the start of TRT was 82.1 months (range, 37-120). The hypogonadal symptoms, the indicators for TRT, were hypodynamia, muscle power decrease, hot flashes, and sexual dysfunction. 

**Table 1:** Baseline parameters (N=20) PSA, prostate specific antigen; LNs, lympho nodes; HDR, high dose rate; EBRT, external beam radiotherapy; Hb, hemoglobin; TRT, testosterone replacement therapy

Parameters	Values
Cancer characteristics	
Initial PSA (ng/ml), mean (range)	15.510 (2.691-50.000)
T stage (%), n (%)	
T2	7 (35.0)
T3a	7 (35.0)
T3b	6 (30.0)
LNs metastasis positive (N1) (%), n (%)	2 (10.0)
Gleason score, n (%)	
6	1 (5.0)
7	5 (20.0)
8	8 (40.0)
9	6 (30.0)
Prostate cancer risk, n (%)	
high	13 (65.0)
very high	7 (35.0)
Kinds of radiotherapy, n (%)	
HDR brachytherapy	19 (95.0)
EBRT	1 (5.0)
Patients' characteristics before TRT, mean (range)	
Age (years)	74.1 (67-85)
Hb value (g/dl)	13.5 (11.8-16.2)
Total testosterone (ng/ml)	0.608 (0.03-2.29)
PSA value (ng/ml)	0.021 (0.006-0.167)
Duration by the start of TRT (months)	82.1 (37-120)
Chief complaints, n (%)	
Hypodynamia	9 (45.0)
Decrease in muscle power	8 (40.0)
Hot flashes	6 (30.0)
Sexual dysfunction	2 (10.0)

TRT continuity

TRT was continued for a median of 31.5 (3-132) months. Twelve (60%) cases could maintain TRT for two years. In the remaining patients, the treatment was discontinued before completion of two years due to ineffectiveness (three cases), symptom remission (three cases), and transfer to another hospital (two cases). No cases showed biochemical and clinical recurrences of prostate cancer. In addition, no adverse effects of TRT were observed in any case, and no patients experienced cardiovascular or thromboembolic events after TRT.

Change in each variable from the baseline to the 24-month visit

**Figure 1:**
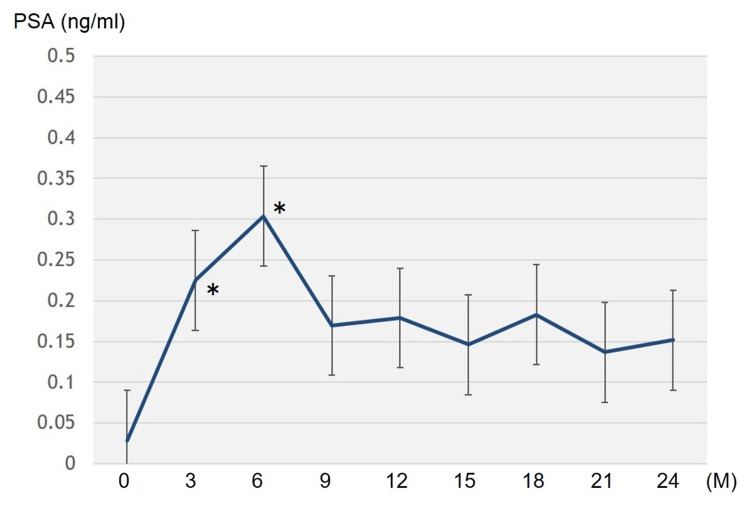
Changes in serum prostate specific antigen (PSA) levels after tesotosterone replacement therapy *Significant difference (p = 0.00287, Z = 2.981 at the third-month visit; p = 0.0121, Z = 2.510 at the sixth-month visit)

The serum PSA levels showed a significant increase for six months following TRT (p < 0.05), and did not change significantly until 24 months (Figure 1).

**Figure 2:**
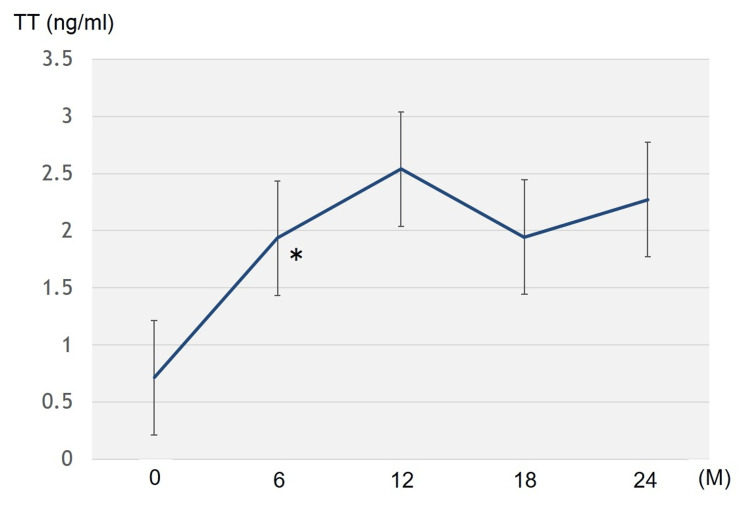
Changes in total testosterone (TT) levels after testosterone replacement therapy *Significant difference (p = 0.00221, Z = 3.059).

No patient showed PSA elevations of 2.0 ng/ml above the nadir (biochemical recurrence). The serum TT levels significantly increased at the 6th-month visit and remained stable until the 24th month (Figure 2).

**Figure 3:**
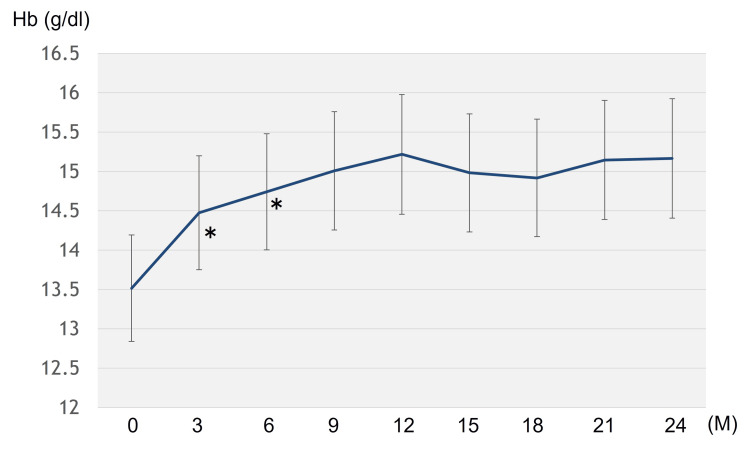
Changes in hemoglobin (Hb) levels after testosterone replacement thrapy *Significant difference (p = 0.00287, Z = 2.981 at the third-month visit; p = 0.0108, Z = 2.550 at the sixth-month visit)

An increase in mean TT levels was observed from 0.714 ng/ml at the baseline to 2.272 ng/ml at the 24-month visit. The Hb levels increased significantly six months after treatment initiation and remained unchanged thereafter (Figure 3).

## Discussion

Here, TRT was performed in 20 patients with prostate cancer who received radiotherapy and continued for a median of 31.5 (3-132) months. No cases displayed biochemical and clinical disease recurrences. Twelve cases (60%) could complete TRT within two years. The serum PSA levels showed a significant increase for six months after TRT, subsequently remaining stable until the 24th month. The transient PSA increase observed during the first six months of TRT may reflect a physiological response to androgen supplementation rather than early tumor recurrence. The stability of PSA thereafter up to 24 months supports this interpretation, suggesting that the initial rise does not necessarily indicate disease progression. These findings suggested good TRT tolerance for 24 months, even in patients with a history of high- or very high-risk prostate cancer. In addition, the 24-month treatment also resulted in significant increases in the Hb and TT levels, confirming that TRT may be biochemically effective.

In 2004, Kaufman and Graydon were the first to report on TRT’s safety in patients following curative radical prostatectomy [13]. Reportedly, none of the seven patients with hypogonadism who received curative radical TRT for a median of 12 months experienced biochemical and clinical recurrences. Other studies demonstrated that TRT in patients who underwent radical prostatectomy [14-16], seed implantation [17,18], HDR brachytherapy, and EBRT [19,20] did not significantly increase biochemical and clinical cancer recurrences. Additionally, in a large population-based observational study from 2014, 1181/149,354 (0.79%) men diagnosed with prostate cancer received TRT after their cancer diagnoses [21]. Moreover, TRT was not associated with increased cancer-specific or all-cause mortality in patients with a prostate cancer history. Furthermore, there was no increased need for salvage ADT, and these results were consistent across stratification by cancer stage, grade, and treatment type. A recent meta-analysis, including 1084 patients after curative local therapy for prostate cancer, demonstrated that TRT did not enhance the biochemical disease recurrence rate [22].

Conversely, evidence on TRT safety for patients with high-risk prostate cancer is currently limited. Some studies included patients with high-risk prostate cancer; however, the population size was small. One study described that TRT did not contribute to a significant increase in the serum PSA levels among 21 men with high-risk prostate cancer and radical prostatectomy history [15]. Another study demonstrated that 26 high-risk patients with cancer, defined by a Gleason score 8 or higher, positive surgical margins, and positive lymph node metastasis after radical prostatectomy, who underwent TRT treatment had lower cancer recurrence rates compared with those who did not receive TRT [16]. Ory et al. reported that two cases experienced biochemical recurrence following EBRT among 30 patients with high-risk prostate cancer treated with local radical therapy [23]. However, the authors concluded that it was unclear whether PSA relapses were related to TRT or caused by the natural progression of the disease. The present study found no cases with a biochemical disease recurrence among 20 patients with high- or very high-risk prostate cancer curatively treated with radiotherapy.

In the present series, TRT was performed in the presence of the prostate in all patients. A previous randomized controlled trial (RCT) assessed the effects of six-month TRT on the prostate tissue through biopsy [24]. TRT significantly increased the serum TT and dihydrotestosterone concentrations compared to the placebo. It did not affect the hormonal levels in the prostatic tissue. Furthermore, some reports provided TRT safety among patients who are on active prostate cancer surveillance [25,26]. In another study, TRT for a median of 2.5 years in men with untreated prostate cancer was not associated with prostate cancer progression and distal metastasis [25]. Kacker et al. also reported that biopsy progression after TRT over three years was not observed in 28 hypogonadal men on active prostate cancer surveillance [26]. These findings support that TRT may be tolerated for cancer control, even in cases with the presence of the prostate, like in our cases, if prostate cancer was curatively treated.

In one report, only 27.5% of the cases experienced improvement in the normal testosterone levels (≥300 ng/dl) at the 48th month after ADT withdrawal among patients treated with ADT for more than 19 months [7]. In Japan, TE injection is the sole testosterone agent approved by the insurance system [2]. This one is a short-acting testosterone. The serum TT levels rapidly peak within a few days of injection and immediately decline to the baseline 14 days after injection. The present study found that the mean TT level at the 24th month visit could not reach the normal range. Conversely, according to previous global reports, the serum T levels could remain stable in the normal range among most of the cases due to the use of long-acting agents or the daily use of gel supplements [18,19,22]. In Japan, the TE administration frequency must be further considered to achieve stably normal TT levels.

Currently, there is little consensus on patient selection, TRT duration, biochemical recurrence definition, and treatment discontinuation criteria. The patient selection criteria also differ based on studies. Patients with PSA values <0.2-0.7 ng/ml following radiotherapy are eligible for TRT [22,27]. The heterogeneity in the timing of TRT initiation following radical therapy has also been observed to range from one month to 17 years [22,27]. Conversely, most studies defined biochemical recurrence in patients treated with radiotherapy using the Phoenix definition (nadir + 2.0 ng/ml) [22,27]. Our study only included patients with high-risk prostate cancer; hence, the inclusion criteria were strictly set as follows: baseline PSA <0.2 ng/ml before starting TRT, and more than two years after the treatment.

This study had many limitations. First, the number of participants was extremely limited, and the observation period was insufficient. The small number of participants precluded further subanalysis for lymph node metastasis and risk classification. In addition, given that our analysis was based on only 20 patients, the statistical power to detect rare adverse events or subtle oncological risks is inevitably limited. Therefore, these results should be interpreted with caution. Additionally, this was a retrospective study, which is inherent to selection bias. The patient selection was largely dependent on the discretion of the attending physicians. Second, objective evaluations like the sexual health inventory for men (SHIM) score or the aging male symptoms (AMS) scale were not utilized while analyzing the hypogonadal symptoms, which limited our ability to effectively evaluate treatment efficacy. In this study, hypogonadal symptoms were evaluated based on subjective physician assessment. The incorporation of validated questionnaires such as the AMS scale or the SHIM score in future studies would enable a more objective assessment of treatment efficacy. On the other hand, the Hb levels increased significantly following TRT, suggesting a certain biochemical effect. Finally, the present study lacked control groups, which is likely to be a major limitation. For a more accurate assessment of TRT safety, control measures should be used to reduce biased outcome assessment. RCTs on TRT for patients with prostate cancer undergoing curative therapy are currently unavailable. Therefore, further prospective studies, including a large number of participants, control group, and long-term observation, are required to reach a more definite conclusion. Regardless of the above-mentioned limitations, the biggest strength of this study is that the study population comprised only patients with high- or very high-risk prostate cancer.

## Conclusions

The present study found that serum PSA levels significantly increased for six months after TRT and subsequently remained stable until 24 months, without biochemical recurrence in any patient. Additionally, the 24-month treatment resulted in significant increases in Hb and TT levels. Evidence on TRT safety for patients with high-risk prostate cancer is currently limited. This study suggests that TRT for at least two years, even in patients with high- or very high-risk prostate cancer who underwent radiotherapy, might be tolerated for cancer control. As this was a retrospective study without a control group, further prospective studies, including a large number of subjects and a control group, are required to reach a more definite conclusion.
